# Supplementary material for: Effects of Sex Class, a Combined Androgen and Estrogen Implant, and Pasture Supplementation on Growth and Carcass Performance and Meat Quality of Zebu-Type Grass-Fed Cattle
Source: Animals (Basel). 2021 Dec 2;11(12):3441. doi: 10.3390/ani11123441 (PMC8698134; doi:10.3390/ani11123441)
Supplement: Supplementary file 1 [file animals-11-03441-s001.zip › animals-1464613-supplementary.pdf]

Supplementary materials

Table S1. Probabilities values for interactions between sex class, implant[a], and supplementation[b] on growth performance of grass-fed Brahman males.

Variables	Sex class x Implant	Sex class x Supplementation	Supplementation x Implant	Three-way interaction
Hip height, cm	0.92	0.51	0.54	0.50
Muscle score [c]	0.37	0.30	0.38	0.80
Frame size score [d]	0.25	0.26	0.95	0.95
Chronological age	0.06	0.53	0.61	0.08
BW at the end of SUPPL period	0.21	0.84	0.58	0.82
BW (endpoint) at shipping [e]	0.71	0.68	0.59	0.07
Time to reaching endpoint	0.14	0.96	0.83	0.25
ADG1 (d 0 – d 163)	0.01	0.92	0.09	0.18
ADG2 (d 0 – d of shipping)	0.01	0.95	0.19	0.31
Adjusted BW[f]	0.71	0.68	0.59	0.33
Adjusted ADG2 [g]	0.13	0.94	0.17	0.96

[a] A hormonal implant containing 24 mg estradiol benzoate 17 ß (EB) and 120 mg of trenbolone acetate (TBA).[b] Supplemented group received pasture supplementation with a poultry litter-based supplement. [c] 1=very heavy muscled, and 5=lightly muscled [19]. [d] 1=very large, and 5=very small [19]. [e] Shipping day was the date of loading cattle from the ranch to the abattoir after reaching the endpoint. [f] Carcass-adjusted final BW was calculated from HCW divided by the average dressing percent across treatments and adjusted by a 4% shrink. [g] Carcass-adjusted ADG2 was calculated from carcass-adjusted final BW, initial BW, and days on feed.

Table S2. Probabilities values for interactions between sex class, implant[a], and supplementation[b] on carcass characteristics of grass-fed Brahman males.

Variables	Sex class x Implant	Sex class x Supplementation	Supplementation x Implant	Three-way interaction
Hot carcass weight	0.93	0.51	0.39	0.23
Hot carcass dressing	0.63	0.12	0.68	0.38
Cold carcass weight	0.77	0.23	0.23	0.26
Cold carcass dressing	0.89	0.02	0.35	0.37
Conformation score [c]	0.35	0.37	0.74	0.36
Finish score [d]	0.44	0.61	0.73	0.94
KPH	0.28	0.04	0.64	0.48
Skeletal maturity [e]	0.48	0.49	0.60	0.98
Lean maturity [e]	0.49	0.38	0.59	0.52
Overall maturity [e]	0.83	0.61	0.60	0.29
Adipose maturity [f]	0.48	0.49	0.90	0.65
Ribeye area	0.81	0.77	0.55	0.29
12th -fat thickness	0.28	0.58	0.79	0.41
Marbling score [g]	0.39	0.16	0.39	0.77
Thigh width	0.54	0.23	0.85	0.42
Pelvic limb length	0.76	0.67	0.14	0.12
Carcass length	0.62	0.42	0.23	0.88
Leg perimeter	0.06	0.38	0.12	0.25
Thoracic depth	0.11	0.95	0.16	0.79

[a]A hormonal implant containing 24 mg estradiol benzoate 17 ß (EB) and 120 mg of trenbolone acetate (TBA). [b]Supplemented group received pasture supplementation with a poultry litter-based supplement.[c]Conformation scores: 1 = Very convex, 2 = Convex, 3 = Rectilinear, 4 = Concave, 5 = Very concave [25]. [d]Finish score: 1 = Extremely abundant, 2 = Abundant, 3 = Medium 4 = Slight, 5 = Scarce [26]. [e]Skeletal, lean, and overall maturity: 100–199: represent immature animals (100 is equal to A00 and 199 is equal to A99); 200–299: represent more mature animals (200 is equal to B00 and 299 is equal to B99) [27]. KPH: Kidney, pelvic and heart fat in percentage. [f]Adipose maturity: 1 = Ivory White, 2=creamy White, 3= light yellow, 4= intense yellow, 5= orange; [g]Marbling scores: 1= abundant; to moderate, 2= small, 3= slight, 4: traces, 5= practically devoid [26].

Table S3. Probabilities values for interactions between sex class, implant[a], and supplementation[b] on individual and combined yield (%) of subprimal cuts[c] of grass-fed Brahman males

Variables	Sex class x Implant	Sex class x Supplementation	Supplementation x Implant	Three-way interaction
Tenderloin	0.13	0.45	0.19	0.58
Rib-eye roll and Striploin	0.24	0.37	0.54	0.30
Knuckle	0.23	0.27	0.97	0.64
Center cut Sirloin	0.75	0.20	0.08	0.77
Bottom (outside) round	0.58	0.06	0.33	0.32
Eye of round	0.59	0.40	0.41	0.88
Top sirloin cap or rump	0.48	0.36	0.19	0.35
Top (inside) round	0.25	0.94	0.70	0.86
Shoulder clod with top blade	0.82	0.56	0.91	0.56
Chuck (mock) tender	0.82	0.21	0.93	0.55
Tri-tip	0.12	0.81	0.92	0.70
Chuck roll	0.96	0.38	0.53	0.62
Heel of round	0.07	0.62	0.11	0.34
Inside skirt, flank, rose meat	0.88	0.41	0.63	0.86
Rib plate	0.12	0.06	0.47	0.24
Bone-in Brisket	0.04	0.81	0.16	0.36
Bone -in Fore shank	0.85	0.75	0.24	0.56
Bone-in Hind shank	0.04	0.99	0.24	0.67
High-value boneless cuts [d]	0.89	0.65	0.36	0.26
Medium-value boneless cuts [e]	0.79	0.88	0.91	0.52
Low-value cuts [f]	0.44	0.54	0.99	0.88
Total cuts [g]	0.85	0.65	0.48	0.16
Trimmable fat	0.91	0.09	0.11	0.21
Clean bone	0.55	0.07	0.93	0.65

[a]A hormonal implant containing 24 mg estradiol benzoate 17 ß (EB) and 120 mg of trenbolone acetate (TBA). [b]Supplemented group received pasture supplementation with a poultry litter-based supplement. [c]Equivalence of names for individual cuts in different countries were reported by Montero *et al*. [28] and yield values are expressed as percentages of the cold carcass weight. [d] High-value boneless cuts: tenderloin + rib-eye roll and strip-loin + center cut sirloin or top sirloin butt +eye of round + top (inside) round +bottom (outside) round + knuckle + tri-tip + heel of round. [e] Medium-value boneless cuts: shoulder clod and flat iron + chuck (mock) tender + chuck roll. [f] Low-value cuts: brisket + inside skirt, flank, flank steak, rose meat and shoulder rose + rib plate + fore shank + hind shank. [g] Total cuts: consists of the sum of the high-, medium-, and low-valued cuts.

Table S4. Probabilities values for interactions between sex class, implant[a], and supplementation[b] on meat quality traits of grass-fed Brahman males

Variables	Sex class x Implant	Sex class x Supplementation	Supplementation x Implant	Three-way interaction
Juiciness [c]	0.82	0.36	0.85	0.70
Amount of connective tissue [d]	0.15	0.29	0.41	0.28
Muscle-fiber tenderness [e]	0.11	0.69	0.19	0.39
Overall tenderness [e]	0.10	0.59	0.32	0.24
Flavor intensity [f]	0.57	0.69	0.96	0.90
WBSF [g]	0.86	0.78	0.66	0.09
Cooking loss	0.15	0.17	0.15	0.21
Cooking time.	0.95	0.12	0.58	0.31

[a]A hormonal implant containing 24 mg estradiol benzoate 17 ß (EB) and 120 mg of trenbolone acetate (TBA). [b] Supplemented group received pasture supplementation with a poultry litter-based supplement. [c] 8-point hedonic scale, where 1 = extremely dry, and 8= extremely juicy. [d] 8-point hedonic scale, where 1= abundant amount of connective tissue, and 8= no connective tissue. [e] 8-point hedonic scale, where 1= extremely tough, and 8= extremely tender. [f] 8-point hedonic scale, where 1= extremely bland, and 8= extremely intense. [g] Warner-Bratzler shear force.
